# Synchronous Malignant Gastrointestinal Neuroectodermal Tumor and SMARCA4-Deficient Undifferentiated Carcinoma With Independent Origins in the Small Intestine: A Case Report

**DOI:** 10.3389/fonc.2021.665056

**Published:** 2021-08-27

**Authors:** Cuimin Chen, Weihua Yin, Xingen Wang, Ping Li, Yaoli Chen, Xianglan Jin, Ping Yang, Huanwen Wu

**Affiliations:** ^1^Department of Pathology, Shenzhen Hospital of Peking University, Shenzhen, China; ^2^ Department of Pathology, Peking Union Medical College Hospital, Peking, China

**Keywords:** gastrointestinal neuroectodermal tumor, SMARCA4-deficient undifferentiated carcinoma, EWSR1, case report, small intestine

## Abstract

**Background:**

Malignant gastrointestinal neuroectodermal tumor (GNET) is a rare malignant mesenchymal neoplasm that commonly arises in the small bowel, stomach or colon. Meanwhile, SMARCA4-deficient undifferentiated carcinoma is a rarely reported entity with highly aggressive behavior that may involve the ovary, lung, gastrointestinal (GI) tract, endometrium and other organs. To our knowledge, we describe for the first time, an extremely rare case of synchronous GNET and SMARCA4-deficient undifferentiated carcinoma with independent origins in the small intestine.

**Case Presentation:**

A 46-year-old woman presented with multiple small intestine masses and underwent surgical resection. Two distinct entities, GNET and SMARCA4-deficient undifferentiated carcinoma, were identified. GNET was composed of epithelioid and spindle cells with clear or eosinophilic cytoplasm arranged in sheets, nest, papillary, fascicular, palisade, rosette like or pseudoalveolar pattern. The neoplastic cells were positive for S-100 and SOX-10. Ewing sarcoma breakpoint region 1 gene (EWSR1) rearrangement was confirmed by fluorescence *in situ* hybridization (FISH), and EWSR1-CREB1 fusion was revealed by next-generation sequencing (NGS). SMARCA4-deficient undifferentiated carcinoma was composed mainly of poorly adhesive rhabdoid cells with eosinophilic cytoplasm arranged in a diffuse pattern. Multifocal necrosis, brisk mitotic figures as well as multinucleated tumor cells were observed. The neoplastic cells diffusely expressed pancytokeratin and vimentin, and was negative for SMARCA4(BRG1). Frame shift mutation of SMARCA4 was detected by NGS.

**Conclusions:**

This is the first report that GNET and SMARCA4-deficient undifferentiated carcinoma occurred simultaneously in the small intestine, with the latter showing multiple involvement of the jejunum and ileum. The potential mechanism underlying co-existence of these two rare malignancies is unknown and need further investigations and concern.

## Background

Malignant gastrointestinal neuroectodermal tumor (GNET) is a rare malignancy mainly arising in the gastrointestinal (GI) tract, previously reported as “clear cell sarcoma-like tumor of the gastrointestinal tract” (1). Histologically, it is characterized by the relatively monomorphic epithelioid and/or spindle tumor cells with clear to eosinophilic cytoplasm arranged in various pattern. Immunohistochemically, the tumor cells were positive for S100, SOX10, vimentin, synaptophysin (Syn) and negative for cytokeratin, HMB45, Melan A, DOG1, chromogranin A. Ultrastructural examination showed features of neural differentiation. Genetically, the Ewing sarcoma breakpoint region 1 gene (EWSR1) rearrangement is the most remarkable feature of GNET. EWSR1-CREB1 or EWSR1-ATF1 gene fusions present in most cases.

The switch/sucrose non-fermenting (SWI/SNF) complex regulate the process of chromatin remodeling and has an important effect on the gene transcription (2). SMARCA4(BRG1) is one of the catalytic subunits of the SWI/SNF complex. Inactivation of SMARCA4(BRG1) has been recently implicated in the pathogenesis of some undifferentiated carcinomas. SMARCA4-deficient undifferentiated carcinoma may occur in the lung, ovary, GI tract, uterus and other organs (3–10). Microscopically, the tumor is mainly composed of poorly adhesive cells with rhabdoid features, often with multifocal necrosis, brisk mitotic figures as well as multinucleated tumor cells. The neoplastic cells may diffusely express pancytokeratin and vimentin, and show complete loss of SMARCA4(BRG1). Molecular analysis showed SMARCA4(BRG1) inactivating mutation.

Up to our knowledge, the simultaneous occurrence of GNET and SMARCA4-deficient undifferentiated carcinoma has never been reported in the literature. Herein, we described for the first time, an extremely unique case of a 46-year-old woman with primary GNET and concurrent SMARCA4-deficient undifferentiated carcinoma in the small intestine.

## Case Presentation

**Figure 1:**
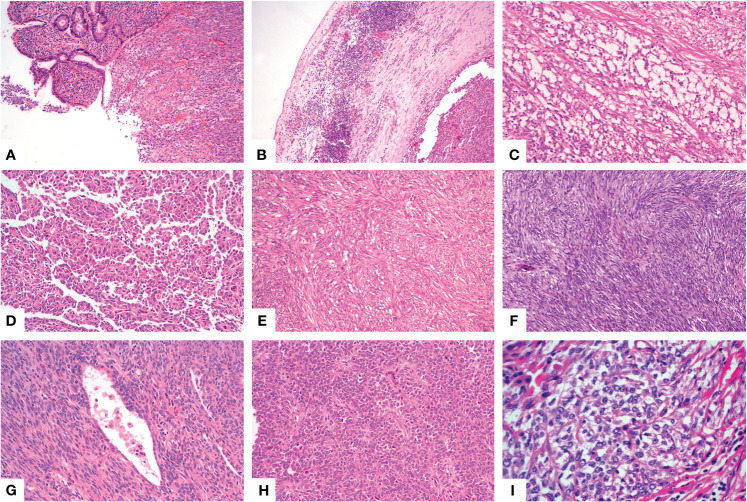
Histological findings of GNET. Low power view shows a sheet like pattern with ulceration on the surface [**(A)**, X100]. Patchy lymphocytic infiltration and multiple lymphoid follicles were observed in the tumor border near the serosa [**(B)**, X100]. The neoplastic cells arranged in microcystic [**(C)**, X200], papillary [**(D)**, X100], fascicular [**(E)**, X100], palisade [**(F)**, X100], pseudoalveolar [**(G)**, X200] or rosette like [**(H)**, X200] pattern with eosinophilic cytoplasm. Some areas showed epithelioid cells with clear cytoplasm [**(I)**, X400].

**Figure 2:**
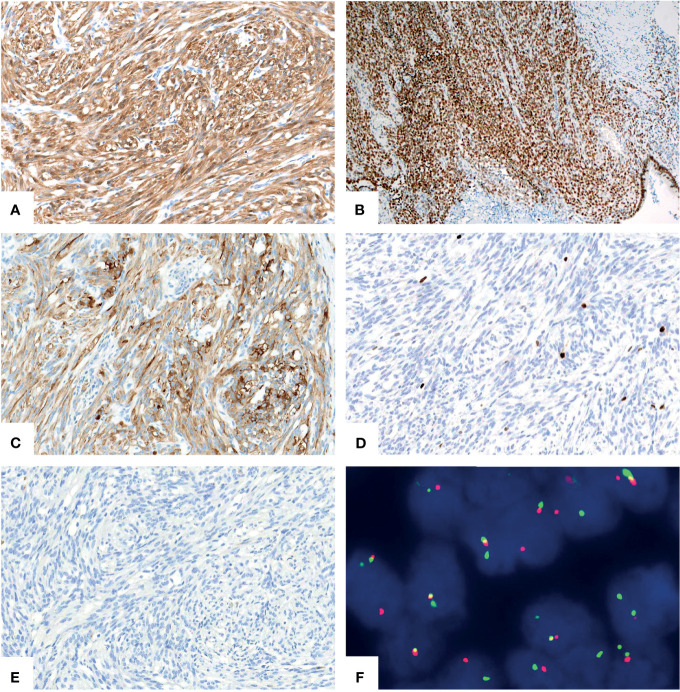
Immunohistochemical and molecular findings of GNET. The neoplastic cells expressed S-100 [**(A)**, X200] and intact SMARCA4(BRG1) [**(B)**, X100]. Syn [**(C)**, X200] was focally positive. The Ki67 [**(D)**, X200] proliferative index was low. Staining for pancytokeratin [**(E)**, X200] was negative. FISH using EWSR1 break apart probe showed red and green split apart signals, suggesting EWSR1 gene rearrangement [**(F)**, X1000].

A 46-year-old woman presented with intermittent abdominal pain and melena for 2 months. Enhanced computed tomography (CT) scan of chest and abdomen revealed a round soft tissue shadows of increased density that was suspected to be located in the small intestine. Additionally, an enlarged lymph node with a maximum diameter of 4.6cm in the right upper mediastinum was suspicious for metastasis. Further small intestine endoscopy suggested a mass about 4 cm in diameter in the small bowel with lumen stenosis. A biopsy was performed and a diagnosis of mesenchymal spindle cell tumor was rendered. A definite diagnosis had not been established due to inadequate sampling. Surgery was performed in our hospital in order to establish a diagnosis and subsequent treatment plan. Intraoperatively, there were multiple masses in the jejunum and ileum. Two segments of jejunum and two segments of ileum were removed. Grossly, a total of seven masses measuring from 1x1x0.5 cm^3^ to 8x4x2cm^3^ were found in the jejunum and ileum, with one in one segment of the ileum, one in the other segment of the ileum, four in one segment of the jejunum, and one in the other segment of the jejunum, respectively. All the masses were completely sampled to histopathological examination. Microscopic examination revealed that one tumor in the ileum was morphologically different from the other six tumors in the jejunum and ileum. The tumor was centered in the muscularis propria with focal extension to the mucosa and ulcer formation (**Figure 1A**). Tumor cells predominantly arranged in sheets, nest, papillary, fascicular and pseudoalveolar architecture with epithelioid and spindle cells. Rosette-like pattern and palisade structure were focally present as well. At high magnification, the tumor cells showed moderate amounts of eosinophilic or clear cytoplasm, ovoid vesicular nuclei with inconspicuous or prominent small nucleoli and infrequent mitotic figures. Necrosis was absent. Peritumoral lymphocytic infiltration and lymphoid follicles were observed (**Figures 1B–I**). Immunohistochemically, the neoplastic cells were strongly and diffusely positive for S-100 and SOX-10, focally positive for Syn, and negative for pancytokeratin, CD117, DOG1, Melan A, HMB45, CD34, ERG, chromogranin A, desmin, and myogenin. INI1 and SMARCA4(BRG1) were stained positively in the nuclei. The Ki-67 index was low (about 5%) (**Figures 2A–E**). Fluorescence *in situ* hybridization (FISH) using EWSR1 break apart probe (Abbott Molecular, Des Plaines, IL) at 22q12 (**Figure 2F**) showed split EWSR1 signals in the tumor cells, indicating the presence of EWSR1 rearrangement. Further next-generation sequencing (NGS) analysis of this tumor in the ileum revealed EWSR1-CREB1 fusion (**Figure 5A**). By a comprehensive evaluation of the microscopy examination, immunohistochemistry and molecular testing, the diagnosis of GNET was established.

**Figure 3:**
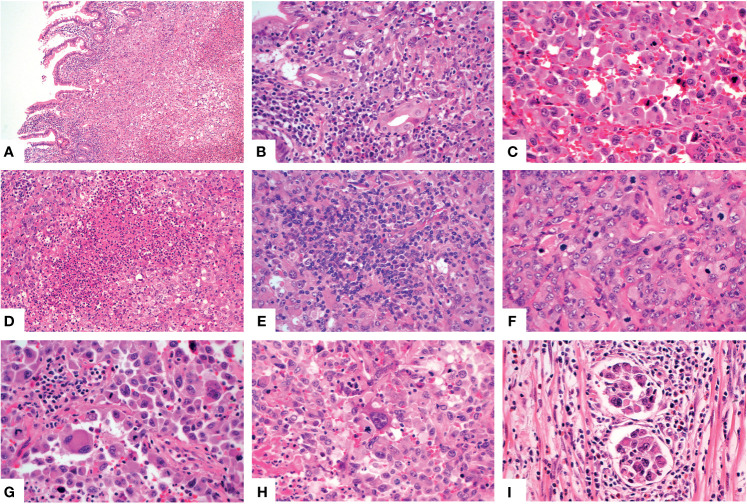
Histologic features of the SMARCA4-deficient undifferentiated carcinoma. The neoplastic cells involved the mucosa [**(A)**, X100]. Higher magnification showed several glands [**(B)**, X400], noncohesive rhabdoid cells [**(C)**, X400], multiple necrosis [**(D)**, X200], patchy lymphocytes and plasma cells infiltration [**(E)**, X400], obvious mitotic figures [**(F)**, X400], multinucleated tumor cells [**(G)**, X400] and large cells [**(H)**, X400]. Multiple lymphovascular permeation was present [**(I)**, X400].

**Figure 4:**
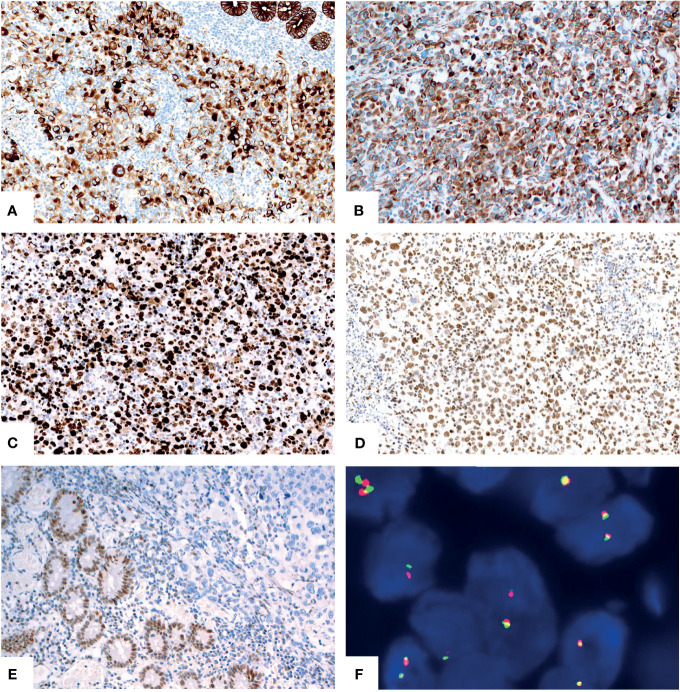
Immunohistochemical and molecular findings of the SMARCA4-deficient undifferentiated carcinoma. The tumor cells diffusely expressed pancytokeratin [**(A)**, X200] and vimentin [**(B)**, X200] with paranuclear accentuation. The Ki67 [**(C)**, X200] proliferative index was high. The tumor showed intact expression of INI1 [**(D)**, X200] and complete loss of SMARCA4(BRG1) [**(E)**, X200, upper right]. No EWSR1 gene rearrangement was revealed by FISH [**(F)**, X1000].

**Figure 5:**
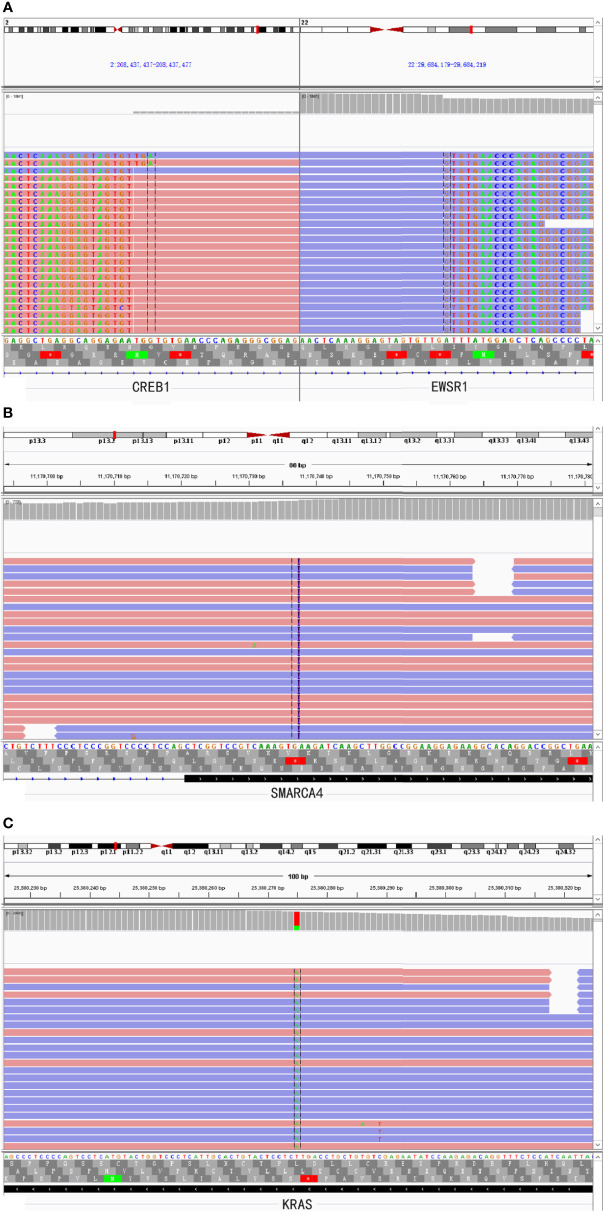
NGS-based comprehensive genomic profiling revealed EWSR1-CREB1 fusion **(A)** in the GNET component and SMARCA4 frame-shift mutation [c.4882_4886dup(p.Lys1630fs)] **(B)** and KRAS hotspot missense mutation [c.183A>T(p.Gln61His)] **(C)** in the SMARCA4-deficient undifferentiated carcinoma component, respectively.

For the other six tumors in the jejunum and ileum, a completely different morphology from GNET was observed. These tumors were located in the upper part of the small intestine, predominantly in the mucosa and submucosa with or without ulcer formation, partially involving the muscularis propria and/or subserosa. Histologically, these tumors were composed of non-cohesive anaplastic rhabdoid cells showing abundant eosinophilic cytoplasm and eccentric vesicular nuclei with one or more nucleoli. Only several neoplastic glandular structures were observed in the mucosa. Multiple necrosis, brisk mitotic figures and patchy infiltration of lymphocytes and plasma cells were observed. Scattered multinucleated tumor cells and irregular large cells were focally noted (**Figures 3A–H**). There was extensive lymphovascular invasion (**Figure 3I**). Immunohistochemically, these tumor cells diffusely expressed pancytokeratin and cytokeratin7, and focally expressed vimentin with characteristically paranuclear accentuation, and was negative for S100, SOX10, desmin, myogenin, chromogranin A, Syn, CD117, DOG1, cytokeratin20, CDX2, TTF-1 and CD34. The Ki67 proliferative index was high (about 90%). Mismatch repair (MMR) proteins, MLH1, PMS2, MSH2 and MSH6, were stained positively in the tumor cells. INI1 protein expression was intact, whereas complete loss of SMARCA4(BRG1) was observed (**Figures 4A–E**). NGS analysis revealed a SMARCA4 frameshift mutation [c.4882_4886dup (p. Lys1630fs)] (**Figure 5B**), which might lead to protein truncation and loss of expression, rather than EWSR1-CREB1 fusion. The absence of EWSR1 rearrangement was also confirmed by FISH using EWSR1 break apart probe (Abbott Molecular, Des Plaines, IL) at 22q12 (**Figure 4F**). All these results supported the diagnosis of SMARCA4-deficient undifferentiated carcinoma for these other six tumors in the jejunum and ileum.

The patient then underwent positron emission tomography-computed tomography (PET-CT) examination. PET-CT scan showed multiple lymph node metastases might occur, including the cervical, supraclavicular, subclavian, mediastinal and abdominopelvic mesenteric lymph nodes. Active metabolized lesions of gluteus maximus, pectineus and adductor brevis were detected, which also suggested the possibility of tumor involvement. In addition, active metabolism in the right parietal lobe that suggested a brain metastasis was observed as well. No subsequent pathological examination was performed. The patient then received chemotherapy. Unfortunately, clinical follow-up revealed that the patient suffered a rapid tumor deterioration and died five months after initial surgical resection.

## Discussion and Conclusions

We described an uncommon case of a middle-aged woman presenting with malignant gastrointestinal neuroectodermal tumor and concurrent SMARCA4-deficient undifferentiated carcinoma with independent origins in the small intestine. To the best of our knowledge, this is the first case report that documents the coexistence of these two rare malignancies in one patient.

GNET was first described by Zambrano et al. (11) in 2003, occurred mainly in the wall of small bowel, stomach and colon in young and middle-aged adults. Microscopically, the tumor was characterized by relatively uniform epithelioid and/or spindle cells with clear to eosinophilic cytoplasm, ovoid vesicular nuclei and inconspicuous or prominent small nucleoli. Variable numbers of mitotic figures can be seen. The neoplastic cells arrange in various patterns, such as solid sheets, nests, papillary, fascicular, pesudoalveolar, rosette like and microcystic architecture. In our case, the palisade structure was presented focally as well. Scattered osteoclast-like giant cells were suggested as a characteristic but not a sensitive morphologic feature of GNET (12, 13). We didn’t find osteoclast-like giant cells in the present case. Genetically, most of the reported GNET cases showed molecular translocation of t(2, 22) (q32.3; q12) resulting in EWS-CREB1 fusion or harbored t(12, 22) (q13; q12): EWS-ATF1 fusions (1, 13). We detected EWSR1 translocation by using FISH and NGS, and further confirmed the diagnosis of GNET in our case.

GNET can be easily misdiagnosed due to its rarity and structural diversity. The differential diagnosis of GNET includes a variety of tumors, such as gastrointestinal stromal tumor (GIST), clear cell sarcoma (CCS), malignant peripheral nerve sheath tumor (MPNST), synovial sarcoma, neuroendocrine neoplasms and melanoma. Comprehensive analysis of morphology, immunohistochemistry and molecular detection is helpful in differential diagnosis.

The prognosis of GNET is not consistent in the literature. Stockman et al. (1) indicated that GNET is a highly aggressive tumor with poor prognosis, while Bin Chang et al. (12) suggested an obviously better prognosis in their case series. Ran Li et al. (13) summarized their own 2 cases and 94 cases in the literature and the statistical analysis showed that GNETs might not be as progressive as described in previous reports, which might be partially due to the improvement of treatment strategies. There were no established standard therapeutic approaches for this neoplasm to date, although sporadic reports showed that some chemotherapy or radiotherapy regimens might be useful to prolong the survival time (12, 14, 15).

SWI/SNF complex functions in chromosome modeling and regulates gene transcription. Subunit alterations of the SWI/SNF complex had been detected in a wide spectrum of human tumors in different organs (16). SMARCA4, SMARCA2, SMARCB1 and ARID1A, which have been extensively studied in previous studies, are subunits of SWI/SNF complex. Inactivating mutations in these subunits can lead to the deficiency of SWI/SNF complex and thus influence cell differentiation, usually associated with undifferentiated histological morphology. Histologically, the tumor cells of SWI/SNF-deficient malignancies in the GI tract usually arranged in a sheet-like pattern, while cribriform, solid and nested/lobular growth pattern may be observed as well. Foci of glandular component or abortive gland formation may be present or not. The neoplastic cells typically, but not always, showed anaplastic medium to large sized cells with rhabdoid morphology, characterized by dyscohesive cells with abundant eosinophilic cytoplasm, eccentric nuclei with vesicular chromatin and obvious nucleoli (3, 4, 8, 9). Some cases showed spindle cells with sarcomatoid differentiation (6). Prominent necrosis, mitotic activity and multinucleation were frequent findings amid the tumor. Extensive permeation of lymphovascular spaces was observed in many cases. The clinical behavior of SWI/SNF-deficient undifferentiated carcinomas is highly aggressive. Researches on new treatments for SWI/SNF complex-deficient carcinomas, including synthetic lethality-based strategies that target BRG1-deficient cancers, are emerging in recent years (17–19).

SMARCA4(BRG1) is one of the catalytic ATPase subunits of the SWI/SNF complex. SMARCA4-deficiency had been reported in the small cell carcinomas of the ovary, hypercalcemic type (5), thoracic malignancies (20), non-small cell lung cancers(NSCLC) (3), sinonasal carcinomas (9), undifferentiated endometrial carcinomas (8), undifferentiated carcinomas in the GI tract (7) and other organs (2, 10). SMARCA4-deficient carcinomas of the GI tract are rarely reported in the literature. Two cases were reported by Agaimy et al. (6) among a series of 13 cases of SWI/SNF complex-deficient undifferentiated/rhabdoid carcinomas of GI tract, while another two cases were reported by Tessier-Cloutier et al. (7). It should be noted that SMARCA4-deficient carcinomas of the GI tract might present with multifocal masses and were easily mistaken for metastatic cancer. In our case, the SMARCA4-deficient carcinoma occurred multifocally in the ileum and jejunum, but no clinical or imaging information suggested the possibility of a primary lesion in any organ other than the small intestine. The SMARCA4-deficient undifferentiated carcinoma has similar histological morphology to other SWI/SNF-deficient malignancies as documented above. Immunohistochemically, the tumor cells usually showed strong reactivity for vimentin and pancytokeratin, but loss of SMARCA4 (BRG1) protein expression. Loss of MMR proteins by immunohistochemistry may occur in some cases without lynch syndrome (6, 7). In addition, primary SMARCA4-deficient carcinomas of the small or large intestine might not express typical intestinal markers such as CK20 and CDX2, and some cases might express CK7 instead. Such immunohistochemical results could not exclude the possibility of a primary tumor in the GI tract. In our case, tumor cells expressed positively for pancytokeratin, cytokeratin7 and vimentin and negatively for cytokeratin20 and CDX2. MMR protein expression was intact. Interestingly, strong and diffuse expression of TFE3 protein was also observed with unknown significance in our case. Subsequent NGS analysis showed a frame-shift mutation of SMARCA4 gene, which might result in SMARCA4(BRG1) protein truncation and inactivation, and the loss of normal nuclear expression. Mutations of KRAS (**Figure 5C**) and TP53 genes were detected by NGS as well. In a previous study, SMARCA4 (BRG1) inactivating mutations co-existed with mutations in KRAS, TP53, LKB1, NRAS and CDKN2A were observed in the cell lines of NSCLC, suggesting potential synergistic mechanisms that may attribute to tumorigenesis (21, 22). Similarly, SMARCA4(BRG1) often co-mutated with TP53, CDKN2A, STK11 and KRAS in human NSCLC samples (22). Both TP53 and KRAS mutations are early and frequent molecular alterations in small bowel adenocarcinoma and may play an important part in tumorigenesis, whereas SWI/SNF complex is crucial for cell proliferation and differentiation (21, 23, 24). We hypothesized that TP53 and KRAS gene mutations might facilitate tumorigenesis and a subsequent loss of SMARCA4(BRG1) might promote tumor dedifferentiation and progression in our SMARCA4-deficient undifferentiated carcinoma. However, the well-differentiated glandular structures in our case were too few to separate them from the undifferentiated area for NGS to identify the similarities and differences between the morphologically distinct areas. The exact mechanism merits further investigations.

Malignant tumors with rhabdoid morphology should be in the list of differential diagnoses of SMARCA4-deficient carcinomas, including epithelioid sarcoma, atypical teratoid/rhabdoid tumor (AT/RT), malignant rhabdoid tumor, melanoma, rhabdomyosarcoma and carcinomas with deficiency of other subunits of SWI/SNF complex.

The coexistence of SMARCA4-deficient carcinomas and other tumors involving the GI tract is extremely rare. Only one case of mixed adeno-neuroendocrine carcinoma in the right colon has been reported to harbor a SAMRCA4 mutation in the neuroendocrine carcinoma component (25). On the contrary, GNET occurred as a secondary malignancy is not uncommon. A subset of case reports has documented that GNET developed after treatment with chemotherapy and/or radiotherapy for the primary tumor, such as hepatoblastoma, neuroblastomas, gastric adenocarcinoma and melanoma (26–30). In our case, the patient had no history of other tumors before and had not received any chemotherapy or radiotherapy.

No morphological overlap between the two components (GNET and SMARCA4-dificient carcinoma) was found in our case, although all tumors were completely sampled. Besides, both gross and microscopic examination showed that the GNET component and the SMARCA4-deficient carcinoma component were completely separated. As mentioned above, immunohistochemical expression and molecular alteration of the two tumors were completely distinct as well. Moreover, no germline mutation was detected in our patient. All the above support that these two tumors were independent of each other, as evidenced by their gross, microscopic and molecular features.

The pathogenesis of the concurrence of these two rare malignancies remains unclear and needs further clarification. It is worth noting that recent researches have suggested the potential association between FET family fusion oncoproteins, of which EWSR1 is one of the members, and SWI/SNF complexes on the tumor development (31, 32). Meanwhile, the interaction of BRG1/BRM-associated factor (BAF) complex with EWSR1, which depends on the EWSR1 prion-like domain, has been found in Ewing sarcoma (33). However, whether there was a possible link between the two tumors of our case or merely an accidental event was unknown.

In summary, we described an extremely case of synchronous GNET and SMARCA4-deficient undifferentiated carcinoma with independent origins in the small intestine of a middle-aged woman. Both tumor components in this case might be easily misdiagnosed. A comprehensive evaluation of the morphologic, immunohistochemical and molecular features of the tumors is essential to make a correct diagnosis. The possible mechanism underlying the concurrence of these two malignancies needs further investigation.

## Data Availability Statement

The original contributions presented in the study are included in the article/supplementary material. Further inquiries can be directed to the corresponding author.

## Ethics Statement

Written informed consent was obtained from the individual(s) for the publication of any potentially identifiable images or data included in this article.

## Author Contributions

CC was responsible for histological diagnosis, literature search, clinical data collection, photo preparation and wrote the manuscript. HW contributed to the NGS detection, pathologic diagnosis and revised the manuscript. WY and XW contributed to the pathologic diagnosis. PL offered pathological help. YC and XJ performed fluorescence *in situ* hybridization. PY participated in the figure preparation. All authors contributed to the article and approved the submitted version.

## Conflict of Interest

The authors declare that the research was conducted in the absence of any commercial or financial relationships that could be construed as a potential conflict of interest.

## Publisher’s Note

All claims expressed in this article are solely those of the authors and do not necessarily represent those of their affiliated organizations, or those of the publisher, the editors and the reviewers. Any product that may be evaluated in this article, or claim that may be made by its manufacturer, is not guaranteed or endorsed by the publisher.

## Abbreviations

GNET, Malignant gastrointestinal neuroectodermal tumor; GI, gastrointestinal; EWSR1, Ewing sarcoma breakpoint region 1 gene; FISH, Fluorescence *in situ* hybridization; NGS, Next-generation sequencing; Syn, Synaptophysin; CT, Computed tomography; PEC-CT, Positron emission tomography-computed tomography; AT/RT, Atypical teratoid/rhabdoid tumor; BAF, BRG1/BRM-associated factor; HE, Hematoxylin and eosin.
